# Revealing the deformation mechanism of amorphous polyethylene subjected to cycle loading *via* molecular dynamics simulations

**DOI:** 10.1039/c8ra05868g

**Published:** 2018-09-18

**Authors:** Qihong Fang, Yuanyuan Tian, Hong Wu, Jia Li

**Affiliations:** State Key Laboratory of Advanced Design and Manufacturing for Vehicle Body, Hunan University Changsha 410082 PR China lijia123@hnu.edu.cn; State Key Laboratory of Powder Metallurgy, Central South University Changsha 410083 PR China hwucsu@csu.edu.cn; Shenzhen Nonfemet Technology Co., Ltd Shenzhen 518122 PR China; School of Materials Science and Engineering, Northwestern Polytechnical University Xi'an 710072 PR China

## Abstract

Despite tremendous efforts being devoted to the study of the deformation behavior of polyethylene, the deformation mechanism of an amorphous polyethylene polymer under cycle shear-loading remains largely unknown. Here, we report the cycle shear deformation mechanism of an amorphous polyethylene polymer using molecular dynamics (MD) simulations. The stress–strain behaviors, including the elastic, yield, strain hardening, and strain softening regions, are qualitatively in agreement with the previous results. The values of the yield stress, Young's modulus and ultimate strength obtained from MD simulations are consistent with the previous data. The effects of the shear strain rate, temperature, and cycle shear-loading number on the stress–strain behaviors are investigated. Higher strain rate and a lower temperature result in a higher strength in the amorphous polyethylene polymer, attributed to the agglomeration of high local strains. With the increase of the cycle shear-loading number, the high strain region gradually expands from the upper and lower surface to the interior of the polyethylene polymer matrix, which provides the origin of crack initiation. The energy contributions are used in elucidating the inherent deformation mechanisms within the elastic, yielding, strain hardening, and strain softening regions, and the variation trend of energy is consistent with the stress–strain response.

## Abstract 2

Despite tremendous efforts being devoted to the study of the deformation behavior of polyethylene, the deformation mechanism of an amorphous polyethylene polymer under cycle shear-loading remains largely unknown.

## Graphical abstract

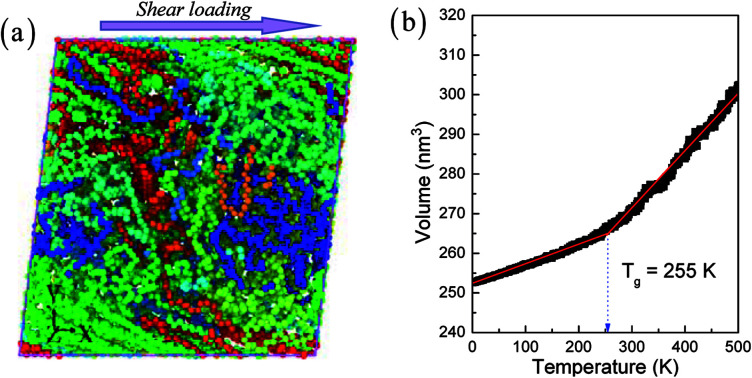

## 1. Introduction

Polymers, due to their good processing performances and excellent mechanical properties, have an important role in industrial materials. The flexibility of tuning a polymer's properties comes from a number of available degrees of freedom: choice of monomers, branching, tacticity, copolymers, blends and composites.^1–3^ Here, the common material polyethylene is selected as the object of study.

Through the computational simulation of polymers, the complex behavior of highly complex polymeric systems can be understood deeply. Coarse grain techniques using united atoms^4–12^ have been widely used to simulate polymer systems and obtain deformation behaviour at different lengths and time scales. A large number of reports have dealt with the plastic deformation within amorphous polymers, where the shear stress drives plastic deformation in glassy polymers to induce structural change.^13^ For example, shear stress could alter the flexing of dihedral angles, leading to the formation of local pockets of liquid-like molecular material.^14^ In line with this notion, shear strain is treated as the accumulation of rotations of kink pairs along the polymer chains and the corresponding model is developed.^15^ In addition, the effect of shear strain on plastic deformation in bulk metallic glasses is revealed during deformation. For instance, by MD simulations, plastic deformation in nanostructure glasses takes place due to local atom rearrangement which produces shear transformation as nucleation sites for additional transformations.^16^ Using molecular statics (MS), the deformation behavior of 3D metallic glasses is studied.^17,18^ In these studies, plastic deformation is due to highly localized heterogeneous atomic rearrangements to generate local stresses. Although studies of atomic/metallic glasses have provided insights into the deformation behavior, the complex bonding of glassy polymers makes their deformation more difficult.

However, so far, numerous computer simulations are performed to study the deformations of glassy polymers in detail. For example, the mechanical deformation of an amorphous linear polyethylene-like polymer glass subjected to uniaxial tension at 100 to 300 K by atomistic Monte Carlo (MC) simulations is studied.^19^ The stress is firstly elastic and then yields with the increase in strain, and finally strain hardening occurs. In the elastic stage, the mechanical work is mainly translated into the nonbonded internal energy. From the yielding stage forward, the intrachain contributions begin to take effect. For the same strain, the stress is larger at lower temperatures with a higher strain rate.^19^ The effect of the constraints and deformation protocol on uniaxial deformation of a polyethylene-like polymer glass by MC simulation is explored.^20^ The study indicates that the constrained bonds and deformation protocol dramatically affect the deformation of polyethylene-like polymer glass, based on the analysis of the stress–strain behavior, evolution of the energy and density, distribution of dihedral angle, and pair correlation function.^20^ The mechanical properties of the polyethylene subjected to uniaxial tension at temperatures of 10 to 500 K are studied by MD simulation.^21^ The results reveal that the value of the Young's modulus decreases with increasing temperature. At low temperatures, the stress–strain response undergoes elasticity, yield, and plastic flow, but it suffers with the viscoelasticity at high temperatures.^21^ Plastic deformation of a typical flexible chain glassy polymer–polypropylene subjected to pure shear is analyzed.^22^ The results indicate that torsional freedom degrees control the conformational changes, due to the inflexible feature in the backbone bonds and bond angles.^22^ In addition, the mechanical response of glassy polymers is studied *via* probing the elastic properties of glassy polypropylene using MS techniques.^23,24^

The previous work mentioned above indicates that the plastic deformation of polypropylene depends on the temperature, and applied strain rate. Moreover, it is worth studying the cyclic deformation of polypropylene subjected to homogeneous stress. The effects of the loading rates, temperatures and cycle loading number on the deformation behaviors of amorphous PE is an important issue, however, it is still not well understood during shear loading deformation. In this study, we investigate the elastoplastic properties and deformation behaviors of amorphous polyethylene under shear loading using MD simulations. The effects of shear strain rates and temperature as well as cycle shear-loading number are considered. The deformation mechanisms are shown through describing chain slipping. In addition, the potential energy of amorphous polyethylene is also depicted to explain the elastoplastic deformation behavior. The paper is organized as follows. The simulated detail is given in Section 2. The results and discussion are described in Section 3. The conclusions based on those observations are summarized in Section 4.

## 2. Simulation methodology

**Fig. 1:**
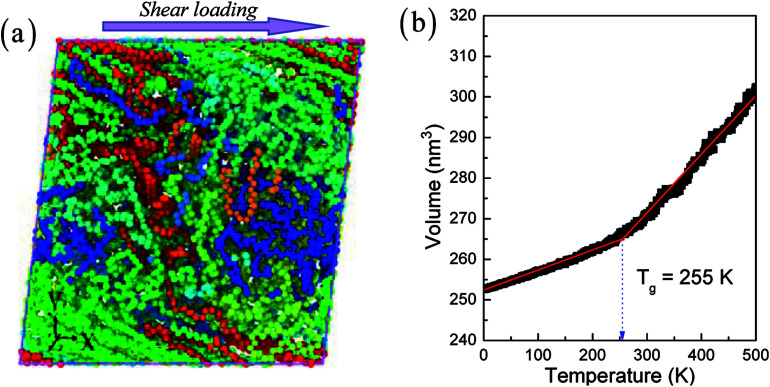
A deformed configuration of amorphous polyethylene (a). The shear loads are applied along the *x*–*y* plane, as indicated by the arrow. The volume *vs.* temperature in polyethylene during the cooling process (b).

Fig. 1 shows amorphous polyethylene deformed during shear loading. The initial structure of amorphous polyethylene is generated by C code, as follows: (1) a single perfect polyethylene is produced based on the atomic coordinate relation; (2) other perfect polyethylenes are obtained based on the first polyethylene by changing the atomic coordinate; (3) the formed polyethylenes are set to a system of 800 K to form the amorphization. Here, the amorphous polyethylene system has large numbers of monomers with 10 000 united atoms. The united atoms stand for full atoms (*i.e.*, CH_2_) to improve computational efficiency.^25–28^ The length and number of chains used in amorphous polyethylene are 1000 monomers and 10, respectively. The Dreiding potential, including bond stretching changes, bond-angle changes, dihedral-rotation changes, and van der Waals nonbonded interactions, is applied on the interatomic force field of a united atom model for polyethylene.^25–28^ The respective parameters of the force field in the amorphous polyethylene system are given in the previous work.^28^Fig. 1b shows the relationship of volume evolution and temperature in polyethylene during the cooling process, where the polyethylene is cooled at a cooling rate of 0.5 K ps^−1^ from 500 K to 0 K based on the previous work.^29^ By the discontinuity point in the slope between the specific volume and temperature,^29^ a glass transition temperature of 255 K can be determined in the amorphous polyethylene system, which agrees with the previous results.^28,29^ Hence, the temperatures of 10, 100, 250, 400, and 800 K are purposely chosen, to study the temperature effect on the shear deformation behaviour.

The periodic boundary conditions are selected along all directions in the current MD simulation, and the time-step is set to 1 fs. According to previous work,^27,28,30^ the system of MD simulation is set at 800 K to generate the amorphous polymer structure, and then cooled to the desired temperature. This relaxation can be achieved by four steps, as follows: (1) firstly, the system of the amorphous polymer is applied to NVT dynamics at 800 K for 100 ps, and then NPT dynamics at 800 K for 500 ps; (2) during the next relaxation, the system of the amorphous polymer is cooled to the desired temperature at a cooling rate of 0.5 K ps^−1^, and then is carried out using the NPT ensemble for 500 ps at the desired temperature for relaxing the system and alleviating the out-of-balance forces and net stresses. The system of the amorphous polymer is deformed at the applied shear strain rates between 1 × 10^6^ and 1 × 10^9^ s^−1^ under the NPT ensemble with a zero-pressure condition, to investigate the shear strain rate effect using the MD simulation.^30–32^ The large-scale atomic/molecular massively parallel simulator (LAMMPS)^33^ is applied to deform amorphous polyethylene. The open visualization tool OVITO^34^ is used for post-processing and visualization.

The stresses in different directions are calculated by the average of all atomic stresses, as follows^35^1
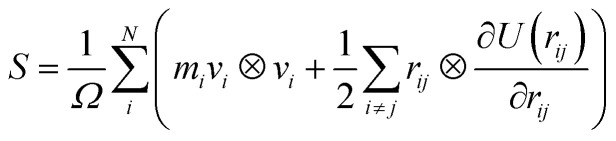
where *S* is the average viral stress with six components, *Ω* is the volume of the cut-off domain, *m*_*i*_ is the mass, *v*_*i*_ is the velocity of the atom *i*, ⊗ is the tensor product of two vectors, and *N* is the total number of atoms in the domain.

## 3. Results and discussion

### 3.1 The stress–strain behaviour

**Fig. 2:**
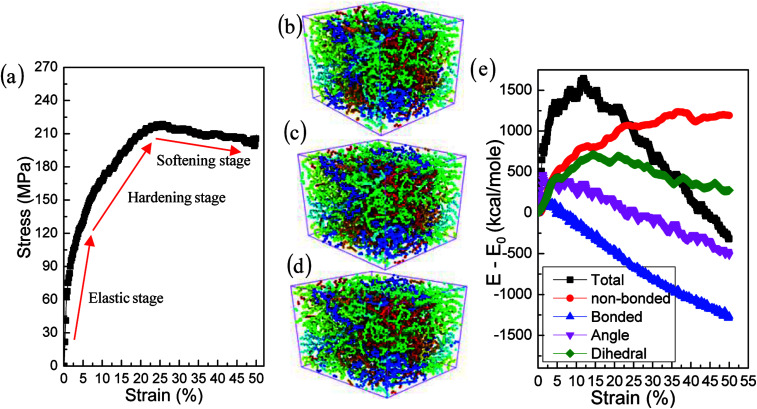
Shear stress *versus* shear strain curves at the shear strain rate of 1 × 10^9^ s^−1^ and a temperature of 250 K (a). The structures of amorphous polyethylene at the shear strain of 30% (b), 40% (c), and 50% (d). Energy decomposition of an amorphous polyethylene system (e).

Fig. 2 presents the stress–strain curve and the structures of amorphous polyethylene at different shear strains. According to classical mechanics, the stress–strain curve can be divided into four distinct regimes: elastic, yield, hardening and softening stages^36–38^ (Fig. 2a). The elastic regime is defined as the region which presents the stress to increase linearly with the increasing strain. This trend is consistent with the deformation behavior of metals.^37^ The stress increases nearly linearly with the increase of shear strain, indicating an elastic deformation. After the yielding, the stress continually increases to a local maximum, suggesting the material hardens. After the hardening stage, the stress reduces with the increase of strain, showing the softening stage in the stress–strain curve. Similar behavior is reported in the plastic deformation of amorphous polyethylene using MD simulations.^28,29,39^

**Fig. 3:**
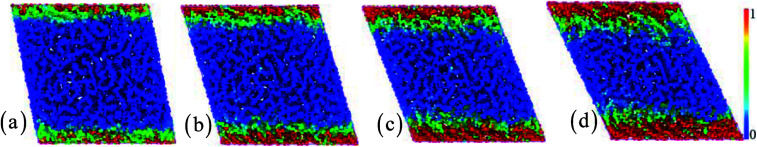
Distributions of local atom shear strain at the shear strain rate of 1 × 10^9^ s^−1^, the temperature of 250 K, and different strains of 20% (a), 30% (b), 40% (c), and 50% (d).

Young's modulus, yield stress, and ultimate strength are the important characterizations of the mechanical properties of materials. The Young's modulus is defined as *σ*/*ε*, where *σ* is the stress, and *ε* is the strain. The yield peak is defined as the maximum stress when the initial plastic deformation just occurs. The ultimate strength is defined as the maximum stress in the stress–strain curve. Fig. 2a indicates that the values of the yield stress, Young's modulus and ultimate strength are 130 MPa, 2.8 GPa and 224 MPa, respectively. These results are in good agreement with the yield stress of 152 MPa, the Young's modulus of 2.5 GPa, and the ultimate strength of 232 MPa in the previous results.^39^ Furthermore, the Young's modulus computed by MD simulations is consistent with an experimental range of between 2.24 and 3.8 GPa.^40^ The corresponding deformation structures are shown in Fig. 2b–d, at the strain of 30%, 40%, and 50%. Fig. 2e illustrates the change of the total energy, the non-bonding energy, the bonding energy, the bond angle energy and the dihedral energy in amorphous polyethylene at the temperature of 250 K and the strain rate of 1 × 10^9^ s^−1^. With the increase of shear strain, the dihedral and non-bonded energies increase sharply at the elastic, yield, and hardening stages, due to chain slippage. When the strain is larger than 25%, the total energy reduces sharply, consistent with the stress–strain response in Fig. 2a. To deeply understand the strain effect, the local atomic shear strain distribution to measure the local inelastic deformation determines the damage location.^41^ The distributions of shear strain are plotted in Fig. 3 at different strains. The high strain regions spread from surface to interior, indicating the increase of damage.

### 3.2 Shear strain rate effect

**Fig. 4:**
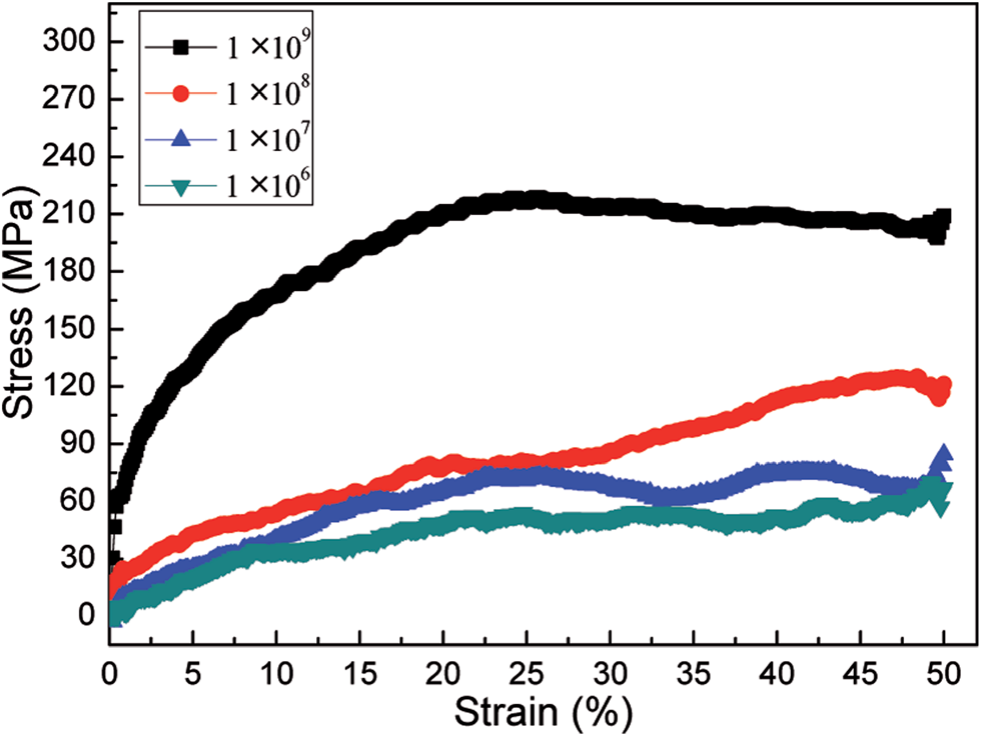
Stress–strain response of amorphous polyethylene deformed at the temperature of 250 K and different strain rates: 1 × 10^6^, 1 × 10^7^, 1 × 10^8^, and 1 × 10^9^ s^−1^.

**Fig. 5:**
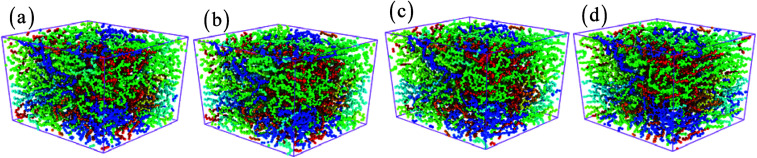
The structure of amorphous polyethylene at the temperature of 250 K and different strain rates: 1 × 10^6^ (a), 1 × 10^7^ (b), 1 × 10^8^ (c), and 1 × 10^9^ s^−1^ (d).

**Fig. 6:**
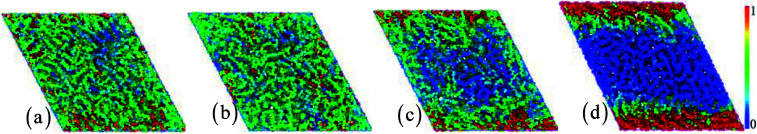
Local atom shear strain distribution at the temperature of 250 K and different strain rate: 1 × 10^6^ (a), 1 × 10^7^ (b), 1 × 10^8^ (c), and 1 × 10^9^ s^−1^ (d).

**Fig. 7:**
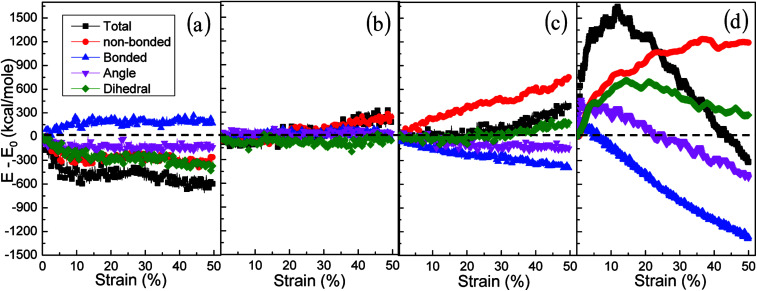
Energy decomposition of an amorphous polyethylene system at the temperature of 250 K and different strain rates: 1 × 10^6^ (a), 1 × 10^7^ (b), 1 × 10^8^ (c), and 1 × 10^9^ s^−1^ (d).

The effects of strain rate on the structural response and mechanical properties of amorphous polyethylene under 250 K are discussed. Fig. 4 shows the relationship of stress and strain in amorphous polyethylene at different strain rates. The yielding strength decreases sharply with the decrease of strain rate, which agrees with the previous results.^42,43^ It suggests that the response of stress and strain is sensitive to the shear strain rate, especially for the high shear strain rate. Fig. 5 shows snapshots of amorphous polyethylene under a shear strain of 50% for different strain rates. It can be seen that the amorphous polyethylene subjected to shear loading deforms by increasing chain length. After shear deformation, the atomic strain distributions at various shear strain rates are shown in Fig. 6. At a low shear strain rate the local strain is evenly distributed, however, at a high shear strain rate the high local strain is located at the end of amorphous polyethylene. This indicates that the microstructural evolution of amorphous polyethylene controls the stress trend (see Fig. 4). In addition, the low strain occurs in the middle region and there is no obvious change in the chain of amorphous polyethylene at high strain rate, leading to the stress oscillation in Fig. 4. The variation in internal energy as a function of strain rate for the amorphous polyethylene system at 250 K is shown in Fig. 7. Here, the energy evolution depends on the applied strain rate, which spans three orders of magnitude. At the low strain rate of 1 × 10^6^ s^−1^, the trends of the total energy and non-bonded interaction energy are very different, compared to that at the high strain rate of 1 × 10^7^ s^−1^. At a strain rate of 1 × 10^7^ s^−1^, the energies associated with bond length and bond angles change slightly with an increase of strain, and the nonbonded interaction energy increases slightly with increasing strain.

### 3.3 Temperature effect

**Fig. 8:**
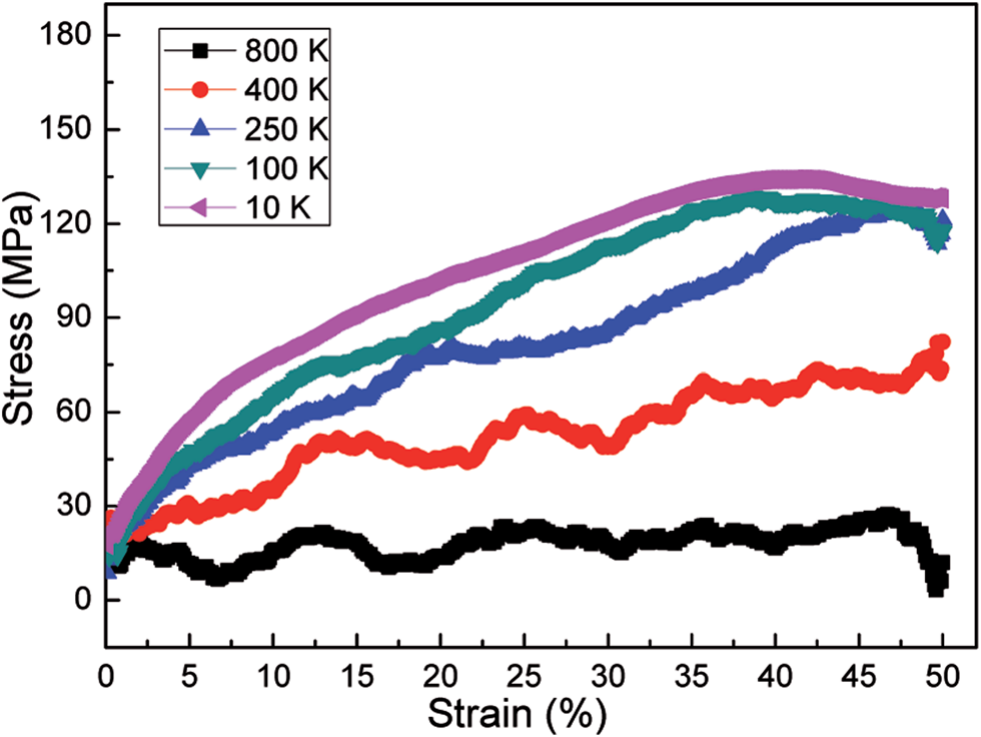
Stress–strain response of amorphous polyethylene deformed at the strain rate of 1 × 10^8^ s^−1^, and different temperatures of 10, 100, 250, 400, and 800 K.

**Fig. 9:**
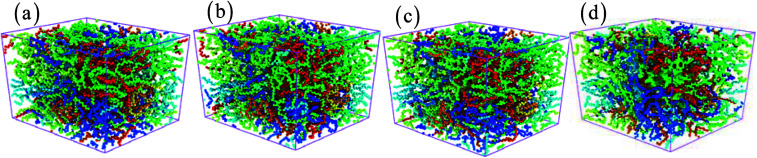
The structure of amorphous polyethylene at the strain rate of 1 × 10^8^ s^−1^, and different temperatures of 10 (a), 100 (b), 400 (c), and 800 K (d).

**Fig. 10:**
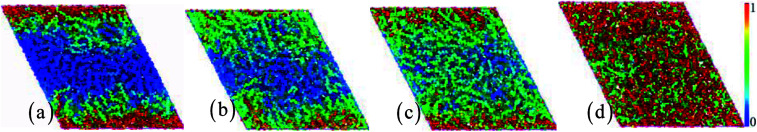
The local atom shear strain distribution of amorphous polyethylene at the strain rate of 1 × 10^8^ s^−1^, and different temperatures of 10 (a), 100 (b), 400 (c), and 800 K (d).

**Fig. 11:**
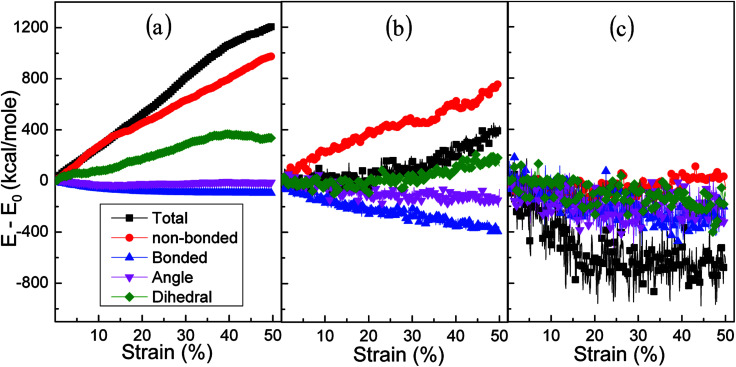
Energy decomposition of an amorphous polyethylene system at the strain rate of 1 × 10^8^ s^−1^, and different temperatures of 10 (a), 250 (b), and 800 K (c).

Temperature plays a key role in the stress–strain response and microstructural evolution of amorphous polyethylene.^44,45^ In Fig. 8, the high temperature can soften the amorphous polyethylene, and the low temperature can strengthen the amorphous polyethylene during shear deformation, similar to the mechanical behaviour of metals at different temperatures. The shear stress of amorphous polyethylene fluctuates when the temperature is larger than 100 K. The structure and local atom shear strain distribution of amorphous polyethylene at the strain of 50% are displayed in Fig. 9 and 10. The high temperature produces the high strain of amorphous polyethylene, to reduce the shear stress. The low temperature inhibits the chain slippage, and the high strain takes place in the small region, resulting in the decrease of the stress fluctuation (see Fig. 4). Fig. 11 shows the change in energy decomposition of an amorphous polyethylene system at different temperatures. The general trend is that the energies associated with bond length and bond angles change obviously with the increase of strain at a wide range from 10 to 800 K. At the high temperature of 800 K, all energies always reduce as the shear strain increases. However, at low temperatures of 10 and 250 K, the total energy, the non-bonded energy, and the dihedral energy increase with the increase of shear strain. The results show that the temperature has a significant effect on the energy evolution, especially at a low temperature of 10 K.

### 3.4 Cycle shear-loading number effect

**Fig. 12:**
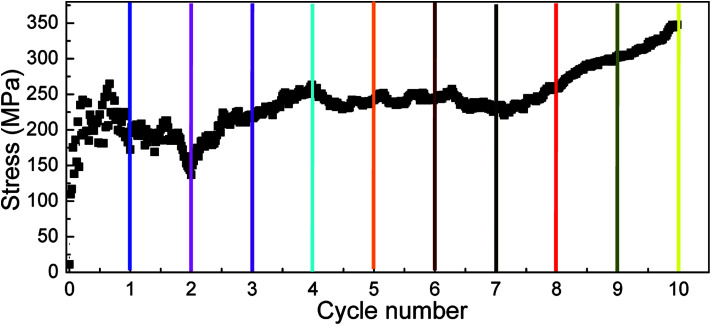
Stress response of amorphous polyethylene deformed at the strain rate of 1 × 10^9^ s^−1^, a temperature of 250 K, and different cycle shear-loading numbers. One shear-loading cycle is defined as the system subjected to one cycle of shear deformation and reverse shear deformation.

**Fig. 13:**
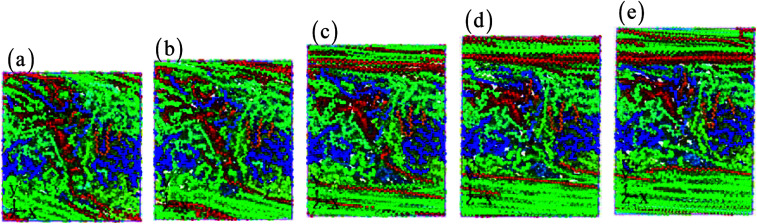
The structure of amorphous polyethylene at the strain rate of 1 × 10^9^ s^−1^, the temperature of 250 K, and different cycle numbers of 1 (a), 2 (b), 5 (c), 7 (d), and 10 (e).

**Fig. 14:**
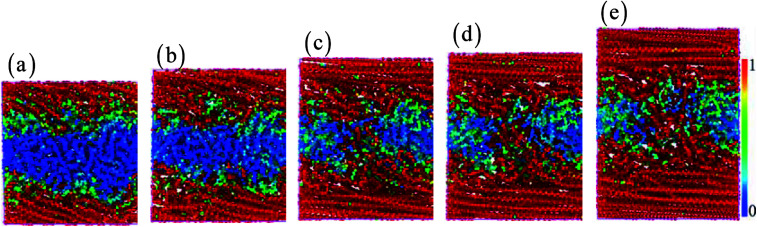
The local atom shear strain distribution of amorphous polyethylene at the strain rate of 1 × 10^9^ s^−1^, the temperature of 250 K, and different cycle numbers of 1 (a), 2 (b), 5 (c), 7 (d), and 10 (e).

**Fig. 15:**
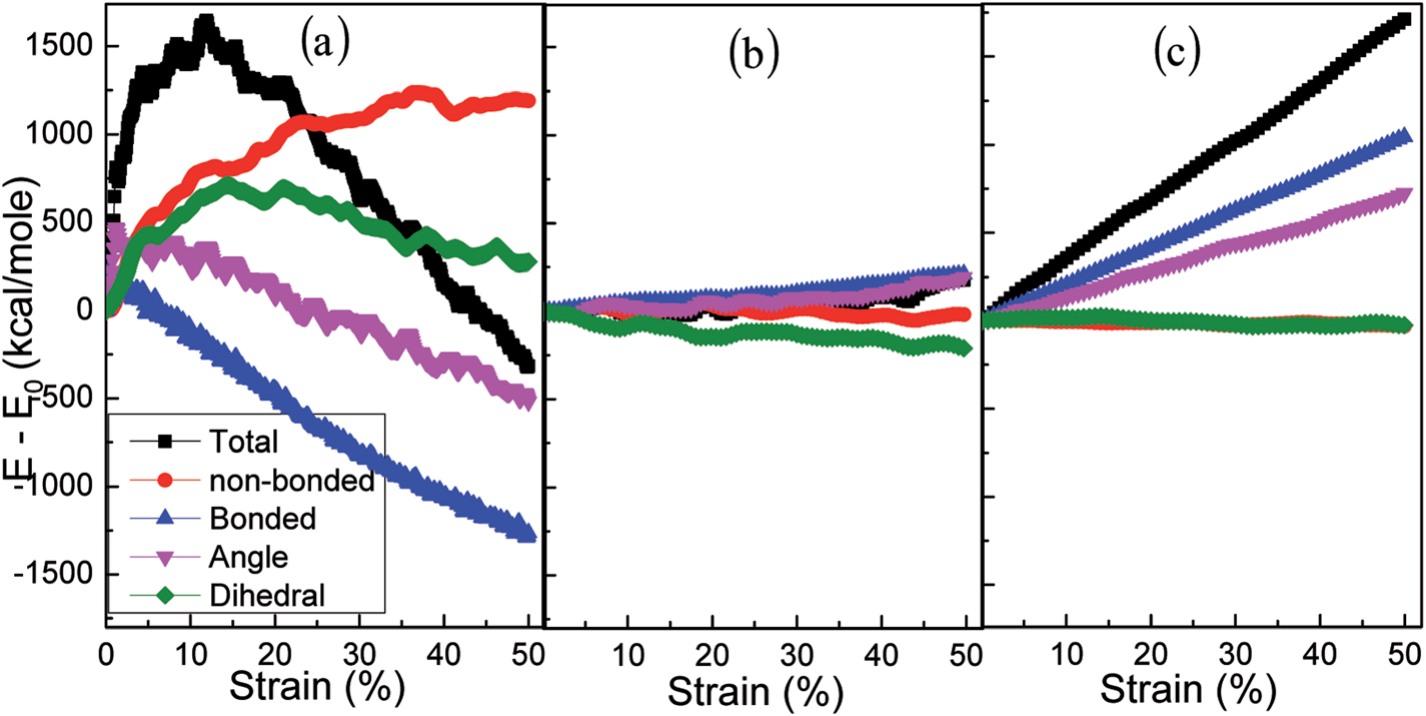
Energy decomposition of an amorphous polyethylene system at the strain rate of 1 × 10^9^ s^−1^, a temperature of 250 K, and different cycle numbers of 1 (a), 5 (b), and 10 (c).

The cyclic shear loading is applied to the amorphous polyethylene,^46,47^ to study the fatigue behavior. The stress sharply fluctuates at cycle numbers below 3, and then keeps stable at cycle numbers from 4 to 8, finally increasing continuously at cycle numbers larger than 9, as shown in Fig. 12. With increasing cycle number, the structure and local atom shear strain distribution of amorphous polyethylene are plotted in Fig. 13 and 14. The chain becomes straight and smooth at the end of amorphous polyethylene, as the cycle number increases. In addition, this trend gradually expands towards the inside of amorphous polyethylene in Fig. 13. At high cycle numbers, a similar shear band occurs through the interior of amorphous polyethylene, resulting in an increase of shear stress. Hence, the evolution of the local atom shear strain distribution as a function of cycle number can provide insight into the accommodation of deformations in amorphous polyethylene. Fig. 15 shows how the energy of amorphous polyethylene evolves as a function of cycle shear number at 250 K. With increasing cycle shear number, the non-bonding energy and the dihedral energy play a weak role in the deformation behaviour. The total energy increases with the increasing strain at high cycle shear-loading numbers, unlike the behavior at the low cycle shear-loading number. Interestingly, all energies change slowly at a middle cycle number due to chain slipping.

Cyclic shear loading with high cycle numbers would result in the fracture of amorphous polyethylene. The strain to the internal amorphous polyethylene increases with the increase of cycle number (see Fig. 14), and finally the cycle maximal stress always exceeds its rupture strength to cause serious failure. In addition, the size of the amorphous polyethylene system strongly affects the fracture mechanism, owing to the size effects on the fracture energy observed in the experiment of polymer fracture.^48,49^ The MD simulations of the fracture could give a fundamental understanding of the solids failure, however, MD simulations are restricted to a certain size and time in the amorphous polyethylene. Recently, the most important development of the multiscale modeling of the fracture or modeling fracture would propose new solutions for the multiscale fracture process.^50,51^ Multiscale methods coupling MD-models to continuum models^50,51^ have been developed, which are conducive to investigating the behaviour of multiscale fracture in the amorphous polyethylene. In future, to deal with the uncertainties of fracture toughness in the amorphous polyethylene, we would use a sensitivity analysis toolbox to quantify the influence of different parameters on uncertain fracture toughness.^52^ The accurate prediction of fracture strength could help to optimize reasonably the design structure of the amorphous polyethylene for industrial applications.

## 4. Conclusions

In the present study, the shear deformations of amorphous polyethylene are investigated by MD simulations. The effects of temperature, shear strain rate, and cycle shear-loading number on the deformation and local atom shear strain distribution in amorphous polyethylene are analyzed in terms of the energy evolution and the stress–strain behaviour. The mechanical properties, including the yield stress, Young's modulus, and ultimate strength, in the amorphous polyethylene can be obtained from MD simulations, which are in quantitative agreement with previous results. The MD simulation reveals that the strength of amorphous polyethylene can be improved by increasing the shear strain rate, or lowering the temperature. The shear strain rate, temperature, and cycle shear-loading number play a key role in the deformation, local atom shear strain distribution and energy evolution. The current study is beneficial for deeply understanding the deformation and strengthening mechanisms of the amorphous polyethylene in complex external environments, and could provide a new study basis for searching for high-performance amorphous polyethylene applied in extreme environments.

## Conflicts of interest

The authors declare no conflict of interest.

## Supplementary Material
